# Medial temporal lobe atrophy, white matter hyperintensities and cognitive impairment among Nigerian African stroke survivors

**DOI:** 10.1186/s13104-015-1552-7

**Published:** 2015-10-30

**Authors:** Rufus O. Akinyemi, Michael Firbank, Godwin I. Ogbole, Louise M. Allan, Mayowa O. Owolabi, Joshua O. Akinyemi, Bolutife P. Yusuf, Oluremi Ogunseyinde, Adesola Ogunniyi, Raj N. Kalaria

**Affiliations:** Division of Neurology, Department of Medicine, Federal Medical Centre Abeokuta, Abeokuta, Nigeria; Institute of Neuroscience, Campus for Ageing and Vitality, Newcastle University, Newcastle upon Tyne, NE4 5PL UK; Department of Radiology, University of Ibadan, Ibadan, Nigeria; Department of Medicine, University of Ibadan, Ibadan, Nigeria; Department of Epidemiology and Medical Statistics, University of Ibadan, Ibadan, Nigeria

**Keywords:** Africa, Brain atrophy, Dementia, Neuroimaging, Nigeria, Stroke, Vascular cognitive impairment, Vascular dementia

## Abstract

**Background:**

Neuroimaging features associated with vascular cognitive impairment have not been examined in sub-Saharan Africans. We determined magnetic resonance imaging (MRI) features associated with cognitive impairment in a sample of Nigerian stroke survivors.

**Methods:**

Stroke survivors underwent brain MRI with standardized assessment of brain volumes and visual rating of medial temporal lobe atrophy (MTA), and white matter hyperintensities (WMH) at 3 months post-stroke. Demographic, clinical and psychometric assessments of global cognitive function, executive function, mental speed and memory were related to changes in structural MRI.

**Results:**

In our pilot sample of 58 stroke survivors (60.1 ± 10.7 years old) MTA correlated significantly with age (r = 0.525), WMH (r = 0.461), memory (r = −0.702), executive function (r = −0.369) and general cognitive performance (r = −0.378). On univariate analysis, age >60 years (p = 0.016), low educational attainment (p < 0.001 to p < 0.003), total brain volume (p < 0.024 and p < 0.025) and MTA (p < 0.003 to p < 0.007) but not total WMH (p < 0.073, p = 0.610) were associated with cognitive outcome. In a two-step multivariate regression analysis, MTA (p < 0.035 and p < 0.016) and low educational attainment (p < 0.012 and p < 0.019) were sustained as independent statistical predictors of cognitive outcome.

**Conclusions:**

Medial temporal lobe atrophy was a significant neuroimaging predictor of early post-stroke cognitive dysfunction in the Nigerian African stroke survivors. These observations have implications for a vascular basis of MTA in older stroke survivors among sub-Saharan Africans.

**Electronic supplementary material:**

The online version of this article (doi:10.1186/s13104-015-1552-7) contains supplementary material, which is available to authorized users.

## Background

Although physical disability is most commonly associated with stroke, cognitive changes and other non-motor consequences are quite frequent in those who survive longer. Up to 64 % of stroke survivors will develop a degree of cognitive impairment and about 30 % succumb to dementia in the long term [1]. In a recent meta-analysis, the pooled prevalence estimates of post-stroke dementia (PSD) within one year of stroke ranged from 7.4 % in population-based studies of first-ever stroke excluding pre-stroke dementia to 41.3 % in hospital-based studies of all strokes including pre-stroke dementia [2]. Despite the potential high burden of vascular cognitive impairment after stroke few pharmacological studies have addressed treatment options [3]. Post-stroke cognitive dysfunction characteristically encompasses a multi-domain impairment of attention and concentration, executive function, language, memory and visuospatial function, with executive function being the earliest and predominantly affected domain [4–6].

There is a large body of structural brain imaging evidence in vascular cognitive impairment (VCI), which suggests that medial temporal lobe atrophy (MTA) or global cerebral atrophy, white matter changes, lacunar infarcts, strategic infarcts and cerebral microbleeds contribute to vascular cognitive impairment, although the relative contributions of each varies across studies [7–13]. However, the neuroimaging substrates of post-stroke cognitive impairment and dementia or VCI have never been examined among sub-Saharan Africans. In studies involving multiracial populations including persons of African ancestry, worse cardio- and cerebrovascular outcomes have often been reported [2]. For instance, in the multiracial South London Stroke Registry (SLSR) Study [14], worse outcome in terms of mortality and higher rates of cognitive impairment and dementia were reported among black participants. Thus, investigating neuroimaging substrates for post-stroke cognitive impairment among sub-Saharan Africans could provide deeper insight into the reasons why people of African ancestry seem to have worse predisposition to poor cerebrovascular outcomes compared to Caucasians and other races. The aim of this study, therefore, was to determine the neuroimaging correlates of VCI 3 months post-ictus in older Nigerian African stroke survivors participating in the Cognitive Function After STroke (CogFAST)—Nigeria Study.

## Methods

### Study design and participants

Stroke patients (≥45 years) were recruited from the stroke registers of two referral hospitals, the Federal Medical Center Abeokuta [15] and University College Hospital, Ibadan in South-western Nigeria between July 2010 and June 2012. Consecutively presenting stroke patients diagnosed by the most senior physician/attending neurologist were admitted to the medical wards of the two specialist hospitals. Subjects and their family/caregivers were approached regarding participation in the study at discharge from hospital or during the initial outpatient visit. Three months after the ictus, 220 stroke survivors were screened for eligibility out whom 145 met the selection criteria for the study but two were further excluded due to incomplete records. The sample for this study consisted of the 58 stroke survivors for whom brain MRI images were available. The inclusion criteria for the study were: 45 years of age or older, duration after stroke within 3 months and clinically confirmed stroke based on history, physical examination and neuroimaging as much as possible. Exclusion criteria were: [1] subarachnoid haemorrhage [2] significant physical illness and motor impairment that precluded paper and computer-based neuropsychological evaluation (e.g. visual impairment, moderate-severe aphasia, hemiparesis affecting the dexterous hand (MRC power grade <3) [3] any co-morbid psychiatric or neurologic illness [4] any systemic disease that could impair cognition e.g. chronic liver disease, chronic kidney disease [5] inability or failure to give consent.

Stroke was defined according to the World Health Organization (WHO) definition [16] and classified using the WHO definition, the Oxford Community Stroke Project Classification (OCSP) [17] and neuroimaging (CT scan and/or MRI) findings,when available. Neuroimaging was not performed on some patients due to limited access and prohibitive cost in Nigeria. The WHO criteria have been shown to have a sensitivity of 73 % for haemorrhage, 69 % for infarction and an overall accuracy of 71 % in Nigeria [18]. The cohort was comprehensively assessed 3 months after stroke, allowing time for the resolution of post-stroke delirium in accordance with the design of Desmond et al. [19]. The evaluation included a medical history, assessment of neurological deficits and MRI scan (n = 58). Cardiovascular risk factors including hypertension, diabetes mellitus, dyslipidaemia, smoking, excessive alcohol use, atrial fibrillation and previous stroke were ascertained from medical history and clinical records. The aggregated vascular risk score was defined as the sum of the number of vascular risk factors identified in each subject (hypertension, diabetes mellitus, dyslipidemia, atrial fibrillation, ever smoked, ever taken alcohol and previous stroke).

Exclusion criteria were: (1) subarachnoid haemorrhage, (2) significant physical illness and motor impairment that precluded paper- and computer-based neuropsychological evaluation (e.g. visual impairment, moderate to severe aphasia, hemiparesis affecting the dexterous hand (MRC power grade <3), (3) any co-morbid psychiatric or neurologic illness, (4) any systemic disease that could impair cognition e.g. chronic liver disease and chronic kidney disease, (5) non-consent to take part in the study. The local research ethics committees granted approval for the study (University College Hospital, Ibadan and Federal Medical Centre Abeokuta), while written informed consent was obtained from each subject. Each subject was informed of the study and possibilities of future publication of the results.

### Cognitive assessment

The neuropsychological instrument consisted of the Community Screening Instrument for Dementia (CSID)—cognitive part [20], the Mini-Mental State Examination (MMSE) previously used among Nigerian cohorts [21] and the vascular neuropsychological battery [1]. The CSID is a paper and pencil test of global cognitive performance which adaptability, validity and utility in populations from different cultural, educational and socio-economic backgrounds have been established [20, 22]. It has a sensitivity of 87 % and specificity of 83 % for the diagnosis of dementia, and has been used reliably and widely to assess cognition in the Yoruba-speaking population of south-western Nigeria, where the present study was conducted [23]. The schedule includes sub-scores for attention, orientation, calculation, short- and long-term memory, language comprehension and expression, praxis and abstract thinking. A raw score method was used for scoring resulting in score range of 0–30 with higher scores indicating better cognitive function. Pre-stroke cognitive status was assessed using the CSID-informant part by trained interviewers.

The Vascular Neuropsychological Battery was devised by us after the NINDS-CSN Harmonization Standards 60-min neuropsychological protocol [1], with minor modifications to ensure adaptability to the language and culture of the study population. The vascular neuropsychological battery consisted of multiple test items examining specific cognitive domains (executive function, memory/learning, language, visuospatial/visuoconstructive skills).

Test items from the Cognitive Drug Research computerized assessment battery were also included in the vascular-neuropsychological battery for the evaluation of attention, processing speed and executive function [the constituent tests included simple reaction time (SRT)—a measure of attention, choice reaction time (CRT)—measuring processing speed, digit vigilance and spatial working memory—measuring attention and working memory, respectively [6]. Further details of the complete cognitive assessment battery are provided as Additional file 1.

### Cognitive diagnosis

To make a cognitive diagnosis on a subject, all available datasets including cognitive scores, functionality and disability scores (the Barthel Index and modified Rankin score) coupled with the physician’s assessment were assembled and discussed by the research team for consensus diagnosis. Functional impairment was defined [24] as a Barthel Index score <75. Final cognitive categorization was based on the VCI criteria proposed by the American Stroke Association/American Heart Association VCI Guidelines [4] and the DSM IV criteria (American Psychiatric Association, 1994).

### Operational definitions of cognitive dysfunction

Failure of an individual subject on a test was defined as a mean score that was at least 1.5 standard deviations below the mean score of the control group. Impairment in a domain was defined as failure on at least 50 % of tests examining that particular domain [25]. Vascular mild cognitive impairment and PSD were defined according to the American Stroke Association/American Heart Association VCI Guidelines [4]. Vascular mild cognitive impairment or vascular cognitive impairment no dementia (vCIND) [4] was defined as impairment in at least 1 cognitive domain (executive function, memory/learning, language, visuospatial/visuoconstructive skills) and normal or mild impairment of activities of daily living independent of motor/sensory symptoms. PSD [4], in accord with the DSM IV criteria, was defined as impairment in ≥2 cognitive domains that were of sufficient severity to affect the subject’s activities of daily living independent of motor/sensory symptoms [4].

### MRI protocol

Brain magnetic resonance imaging (MRI) was performed on a subset of stroke survivors (n = 58) using two MRI scanners operated between 0.2 and 0.35 T. Axial spin-echo T2-weighted (T2W) images (echo time, 80–120 ms; repetition time, 4000–6500 ms; slice thickness, 5 mm); and axial, sagittal and coronal spin-echo T1-weighted (T1W) images (echo time, 9–15 ms; repetition time, 350–500 ms; slice thickness, 5 mm) were acquired. These were complemented by fluid-attenuated inversion recovery (FLAIR) sequences (echo time, 90–120 ms; repetition time, 6000–9000 ms; inversion time, 2000–2200 ms; slice thickness, 5 mm) to allow for better separation and identification of WMHs and cerebrospinal fluid, as used in a previous study [26]. All images were transferred to computer workstation with Clear canvas DICOM viewer and evaluated by two experienced neuroradiologists. All ratings were performed by consensus agreement.

### Image assessment

**Fig. 1:**
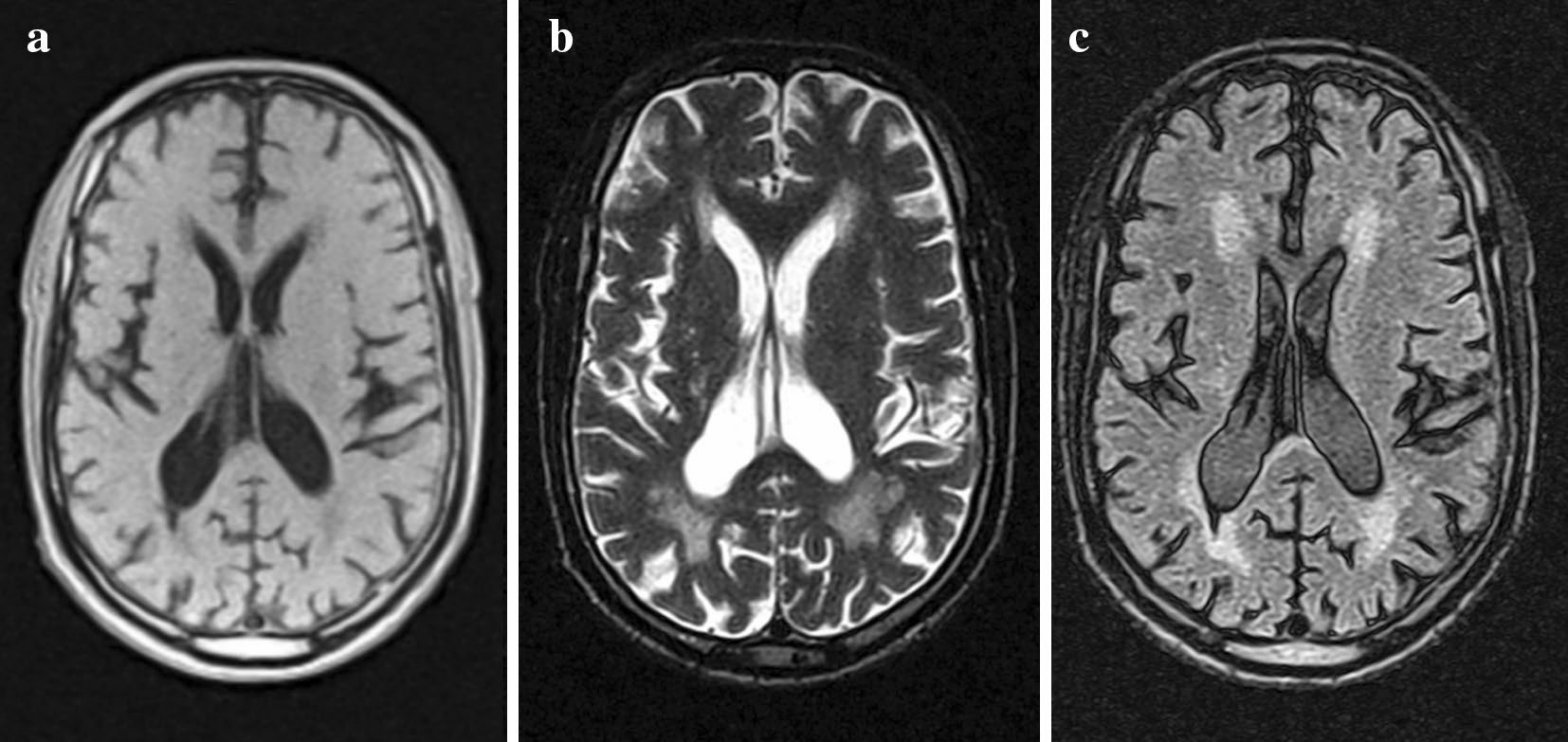
Magnetic resonance imaging (MRI) T1- and T2-weighted (**a**, **b**), and fluid-attenuated inversion recovery (**c**) axial images from a 69-year old male Nigerian stroke survivor showing moderate white matter hyperintensities

**Fig. 2:**
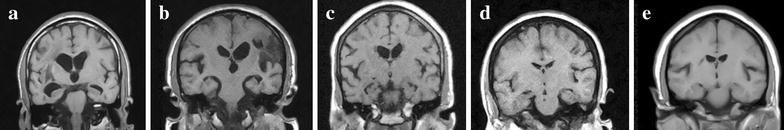
Magnetic resonance imaging (MRI) T1-weighted coronal images showing different degrees of medial temporal lobe atrophy (MTLA) in Nigerian stroke survivors: **a** Grade 4 MTLA in a 58 year old male; **b** Grade 3 MTLA in an 72 year male; **c** Grade 2 MTLA in a 60 year female; **d** Grade 1 MTLA in an 59 year male; **e** Grade 0 MTLA in an 49 year female

White matter changes were assessed using the Scheltens visual rating scale for white matter hyperintensities (WMH) [27]. Ratings were performed on MRI images on computer screen with T2 and FLAIR images. Periventricular WMH score was compiled as a summation of all three periventricular WMH scores in the frontal and occipital regions, as well as along the ventricles; the deep WMH score was a summation of all the deep WMH scores in the four regions assessed (Fig. 1 shows moderate white matter hyperintesities in axial brain MRI images from a 69 year old male stroke survivor in the cohort). The total WMH score for each patient was the sum of all ratings. Medial temporal lobe atrophy (MTA) was evaluated using the Scheltens MTA visual rating scale [28]. Both sides were assessed and the score of the more affected side was used in cases of severe asymmetry. Figure 2 illustrates different degrees of MTA in selected stroke survivors of different ages from the study cohort. Total brain volume (TBV) was measured from the T1-weighted axial images. Slice-to-slice variations in intensity were first removed. This was performed by creating a mask using the brain extraction tool (Bet) from the FSL software (www.fmrib.ox.ac.uk/fsl/). The mean intensity within the mask was determined on each slice, and the overall intensity for the whole slice scaled accordingly. We then used the segmentation tool in SPM8 (www.fil.ion.ucl.ac.uk/spm/) to generate gray and white matter segmentations. A brain mask was generated from the sum of gray + white matter. This brain mask was visually inspected, and manually edited, where necessary, to remove non-brain tissue; total brain volume was measured from the number of voxels in the mask. Total intracranial volume (ICV) was measured from the T2 weighted axial images in a similar fashion, correcting for slice intensity variations, using SPM to segment the brain, then manually editing the segmentation, where appropriate. Total intracranial volume was then taken as the sum of gray matter + white matter + CSF. Ventricular volume was measured from the T2-weighted axial images. We used a previously-created standard space template of probable location of the ventricles in older people [29]. This template was transformed from standard space to each subject and used to mask the CSF segmentation from the previous step. The resulting ventricle segmentation was manually edited, and volume determined. All neuroimaging evaluations were undertaken with the assessors blind to clinical information.

### Statistical analysis

Data were analyzed using the Statistical Package for Social Sciences version 19.0 (SPSS Chicago Inc.). Categorical variables were examined and summarized in percentages, while continuous variables were described using measures of central tendency (mean, median and semi-interquartile range) and compared using the Student’s *t* test, analysis of variance (ANOVA) and Kruskal–Wallis Test. Correlations were examined using Pearson’s correlation coefficient, while logistic regression models were fitted to determine univariate and multivariate relationships between cognitive status and patient-related demographic and neuroimaging variables. Multivariate logistic regression analysis was performed by incrementally feeding demographic and neuroimaging variables which were significant (p < 0.05) in univariate analysis into multivariate analysis in each of three models: model I (Normal vs vCIND); model 2 (vCIND vs PSD) and model 3 [Normal vs (vCIND + PSD)]. Age and years of educational attainment were entered as dichotomous measures and other determinants as continuous measures in the regression models. Age and sex were included in the multivariate model, even if not significant. Unadjusted and adjusted odds ratios (OR) with 95 % CIs were estimated. Level of statistical significance was set at p < 0.05.

## Results

### Participant characteristics

Out of a total of 143 stroke survivors evaluated at baseline 3 months after stroke over the study period, 58 (41 %) had a brain MRI performed in addition to clinical and neuropsychological assessment. Given a significance level, α = 0.05 and assuming a moderate effect size Cohen’s = 0.4, using the G*Power software, the computed power (1 − β) = 0.7599.

Table 1 shows the demographic, clinical and neuroimaging characteristics of those who had MRI and constituted the study group. Subjects who had brain MRI (n = 58) did not differ significantly from those who did not (n = 85) with respect to mean age (p = 0.453); gender (p = 0.302) years of formal education (p = 0.150), stroke type (p = 0.08) and OCSP classification (p = 0.211) (Additional file 2: Table S1). Among subjects who had brain MRI, 6 (10.3 %) subjects had significant pre-stroke cognitive impairment from the informants’ rating of subjects’ cognitive function. Additionally, of subjects who had brain MRI, 26 (44.8 %) subjects had no vCIND, while 24 (41.4 %) and 8 (13.8 %) had vCIND and PSD, respectively based upon our operational criteria.

**Table 1:** Demographic, clinical and neuroimaging characteristics of subjects (N = 58)

Age at baseline: (mean ± SD) years	60.1 ± 10.7
Sex: n (%) female	28 (50)
Total number of years of education:(mean ± SD) years	8.6 ± 5.6
CSID total score (mean ± SD)	24.8 ± 4.6
MMSE score (mean ± SD)	23.5 ± 5.9
Executive function score (mean ± SD)	10.6 ± 4.6
Memory score (mean ± SD)	29.6 ± 10.4
Simple reaction time (mean ± SD)	947.5 ± 861.0
Choice reaction time (mean ± SD)	1170.6 ± 763.4
CESD score (mean ± SD)	6.5 ± 5.4
Stroke type (diagnosed by CT and/or MRI)	
Ischaemic	50 (86.2)
Haemorrhagic	8 (13.8)
Cardiovascular risk factors, n (%)	
Hypertension	53 (91.4)
Diabetes mellitus	13 (22.4)
Dyslipidemia	6 (10.3)
Atrial fibrillation	1 (1.7)
Ever smoked	15 (25.9)
Ever taken alcohol	28 (48.2)
Previous stroke	7 (12.1)
Imaging volumetrics (mean ± SD)*	
ICV (mls)	1331.0 ± 146.7
TBV (mls)	1024.9 ± 132.2
Ven vol (mls)	44.7 + 19.3
TBV/ICV	0.77 ± 0.06
Ven vol/TBV	0.04 ± 0.02
MTA (L + R) total score	7.06 ± 1.67
Vascular lesions on MRI	
Large vessel infarct-right [n (%)]	3 (5.1)
Large vessel infarct-left [n (%)]	3 (5.1)
Frontal infarct-right [n (%)]	4 (6.9)
Frontal infarct-left [n (%)]	3 (5.1)
Parietal infarct-right [n (%)]	17 (29.3)
Parietal infarct-left [n (%)]	13 (22.4)
Basal ganglia small vessel disease-right [n (%)]	15 (25.9)
Basal ganglia small vessel disease-left [n (%)]	9 (15.5)
Total brain WMH (median, semi-interquartile range)^x,^ **	7.00 (0–13.75)
Periventricular WMH (median, semi-interquartile range)^x,^ **	3.00 (0–5.00)
Deep WMH (median, semi-interquartile range)^x,^ **	4.00 (0–9.25)

Mean ± SD is stated for continuous variables and n (%) for dichotomous variables

*MRI* Magentic Resonance Imaging, *ICV* Intracerebral Volume, *MTLA* Medial Temporal Lobe Atrophy, *TBV* Total Brain Volume, *Ven Vol* Ventricular Volume, *WMH* White Matter Hyperintensities, *CSID* Community Screening Instrument for Dementia, *MMSE* Mini Mental State Examination, *CESD* Centre for Epidemiologic Studies Depression Scale

* Volumetric analysis was done only in 54 cases; ** computed based on Schelten’s WMH scale

^x^Data non-normally distributed

### Characteristics of cognitive sub-groups of subjects

Table 2 presents the demographic, cognitive and MRI imaging characteristics of cognitive sub-groups of the cohort, demonstrating the pattern of performance on tests of general cognitive functioning as well as in specific domains of memory (V-NB memory score), executive function (V-NB executive score), attention (SRT), information processing speed (CRT) and mental flexibility (SPMRT). There were statistically significant differences in performance (mean and standard deviation) across the spectrum of stroke survivors (Normal, vCIND and PSD) on each cognitive test.

**Table 2:** Characteristics of cognitive sub-groups of subjects (N = 58)_

Variable	Normal (N = 26)	Vascular CIND (N = 24)	PSD (N = 8)	p value (ANOVA)
	Mean (SD)	Mean (SD)	Mean (SD)	
Age (years)	54.9 (7.8)	62.8 (8.9)	68.3 (15.6)	*0.001*
Education (years)	11.3 (4.1)	6.9 (6.2)	4.6 (4.1)	*0.001*
SRT (ms)	599.1 (622.6)	1001.9 (656.5)	1886.4 (1283.3)	*<0.001*
CRT (ms)	826.3 (442.9)	1296.8 (794.8)	1899.2 (920.9)	*<0.001*
SPMRT (ms)	2186.0 (1068.8)	2827.2 (1542.9)	3741.2 (2850.9)	0.06
Log_ICV	6.12 (0.05)	6.12 (0.05)	6.10 (0.04)	0.414
Log_TBV^a^	6.02 (0.05)	6.00 (0.04)	5.93 (0.06)	*0.001*
Log_Venvol	4.56 (0.17)	4.66 (0.23)	4.66 (0.13)	0.172
TBV/ICV^b^	0.79 (0.04)	0.77 (0.06)	0.70 (0.09)	*0.001*
MTA total (L + R) score^c^	6.28 (1.49)	7.79 (1.58)	8.00 (1.27)	*0.001*
Total WMH score	6.80 (7.53)	11.52 (11.78)	14.57 (15.34)	0.273^d^
Periventricular WMH score	2.38 (2.21)	3.31 (2.38)	3.85 (2.73)	0.231
Deep WMH score	4.42 (6.20)	8.21 (10.11)	10.71 (13.62)	0.492^d^

* p value = significant p values are in italics

^a^Normal vs vCIND, t = 1.209 p = 0.233; vCIND vs PSD, t = 3.160 p = 0.004; normal vs PSD, t = 3.596 p = 0.001

^b^Normal vs vCIND, t = 1.762 p = 0.085; vCIND vs PSD, t = 2.216 p = 0.036; normal vs PSD, t = 4.053 p < 0.001

^c^Normal vs vCIND, t = −3.244 p = 0.002; vCIND vs PSD, t = −0.296 p = 0.770; normal vs PSD, t = −2.608 p = 0.014

^d^Kruskal–Wallis test

Regarding neuroimaging metrics, total intracranial volume (F = 0.898, p = 0.414) and ventricular volume (F = 1.823, p = 0.172) were similar across the subgroups, whereas total brain volume (F = 7.686, p = 0.001) and the ratio of total brain volume to intracranial volume (F = 7.950, p = 0.001) were significantly reduced in cognitively impaired and demented stroke survivors. Medial temporal lobe atrophy (MTA) scores were significantly increased in cognitively impaired and demented stroke survivors (F = 6.776, p = 0.003), while WMHs also showed a similar increasing trend, although this did not attain statistical significance (p > 0.05).

### Correlation of clinical, cognitive and neuroimaging variables

Age correlated significantly with total brain volume (r = −0.393, p = 0.004), MTA total score (r = 0.525, p < 0.001) but not WMH total score (r = 0.206, p = 0.144). Number of years of educational attainment correlated significantly with total brain volume (r = 0.324, p = 0.018) but not MTA (r = 0.263, p = 0.065) or total WMH (r = −0.012, p = 0. 935). MTA correlated significantly with total WMH score (r = 0.461, p = 0.002), total CSID score (r = −0.378, p = 0.019), memory (r = −0.702, p < 0.001) and executive function (r = −0.369, p = 0.016) but not total brain volume (r = −0.203, p = 0.157). Deep WMH frontal score correlated significantly with MTA (r = 0.352, p = 0.013), executive function (r = −0.350, p = 0.013) choice reaction time (r = 0.345, p = 0.015) and memory (r = −0.333, p = 0.021). Deep WMH parietal score correlated with memory (r = −0.502, p < 0.001) and executive function (r = −0.315, p = 0.026), while deep WMH temporal score correlated with executive function (r = −0.303, p = 0.033) but not with memory (r = −0.226, p = 0.123). Pre-stroke informant cognitive score showed significant correlation with post-stroke memory score (r = −0.321, p = 0.022) and a trend with post-stroke general cognitive functioning CSID total score (r = −0.248, p = 0.071). Presence of hypertension correlated significantly with total WM score (r = 0.361, p = 0.001) and total deep WM score (r = 0.375, p = 0.007). The aggregated vascular risk factor load correlated significantly with the female gender (r = 0.372, p = 0.005) but showed a trend with age (r = 0.251, p = 0.064) and MTA (r = −0.248, p = 0.086). Left parietal infarcts were also significantly associated with cognitive dysfunction as an outcome (r = 0.780, p = 0.002).

### Univariate determinants of cognitive outcomes

Table 3 presents univariate logistic regression analyses of statistical predictors of cognitive impairment in three different models. In model I (Normal vs vCIND), education <7 years, and MTA rating were significantly associated with vCIND. In model II (vCIND vs PSD), TBV was significantly associated with PSD. In model III, [Normal vs (vCIND + PSD)], age >60 years, educational attainment <7 years, TBV and MTA significantly differentiated normal (no vCIND) from cognitively impaired (vCIND + PSD) study subjects.

**Table 3:** Univariate logistic regression model of demographic and imaging determinants of cognitive dysfunction among subjects

	Normal vs vCIND			vCIND vs PSD			Normal vs (vCIND + PSD)		
	OR	95 % CI	p value*	OR	95 % CI	p value*	OR	95 % CI	p value*
Age >60 years	3.21	0.98–10.45	0.053	2.54	0.42–15.21	0.308	3.97	1.30–12.13	*0.016*
Female gender	2.24	0.72–6.95	0.163	1.19	0.23–6.17	0.835	2.34	0.81–6.74	0.116
Education <7 years	6.67	1.92–23.18	0.003	3.50	0.37–33.56	0.277	8.52	2.58–23.12	*<0.001*
Total WMH score	1.06	0.97–1.13	0.123	1.02	0.95–1.09	0.580	1.06	1.00–1.13	0.073
Periventricular WMH score	1.20	0.92–1.57	0.181	1.10	0.76–1.60	0.610	1.22	0.96–1.56	0.102
Deep WMH score	1.06	0.98–1.15	0.145	1.02	0.95–1.10	0.600	1.07	0.99–1.15	0.094
Log_ICV	0.22	0.001–5462.00	0.809	0.02	0.001–10,385.0	0.247	0.01	0.001–1144.1	0.439
Log_TBV	0.04	0.01–137.28	0.230	0.03	0.01–0.023	0.024	0.04	0.01–0.20	*0.025*
Log_VenVol	13.44	0.52–347.02	0.117	1.22	0.020–74.90	0.924	18.6	0.81–429.36	0.067
MTLA rating	1.91	1.19–3.06	0.007	1.10	0.59–2.07	0.759	2.05	1.28–3.27	*0.003*

*vCIND* vascular cognitive impairment no dementia, *PSD* post-stroke dementia, *CSID* Community Screening Instrument for Dementia, *MMSE* Minimental State Examination, *V-NB* vascular neuropsychological battery, *ICV* intracranial volume, *TBV* total brain volume, *VenVol* ventricular volume, *MTA* medial temporal lobe atrophy rating, *WMH* white matter hyperintensity, *OR* odds ratio, *CI* confidence interval

* p value = significant p values are in italics

### Multivariate determinants of cognitive outcomes

Demographic and significant univariate neuroimaging predictors were fed into the three models and following which, educational attainment <7 years and MTA rating remained significant independent statistical predictors of post-stroke vascular cognitive impairment no dementia (model I) and of post-stroke cognitive dysfunction (model III) accounting for up to 49 % of the variance of cognitive outcome (Table 4). There were also no differences in the outcomes of the logistic regression analyses with and without cases with pre-stroke cognitive decline (Additional file 2: Table S2).

**Table 4:** Multivariate logistic regression model of significant univariate determinants of cognitive dysfunction among subjects

Variable	Normal vs vCIND			vCIND vs PSD			Normal vs (vCIND + PSD)		
	OR	95 % CI	p value*	OR	95 % CI	p value	OR	95 % CI	p value*
Nagelkerke R^2^	R^2^ = 0.414			R^2^ = 0.470			R^2^ = 0.490		
Age >60 years	1.06	0.19–5.92	0.945	0.50	0.05–5..45	0.579	0.79	0.15–4.27	0.787
Female gender	1.42	0.33–6.17	0.641	0.25	0.02–3.86	0.322	0.83	0.14–4.79	0.834
Education <7 years	6.22	1.35–28.73	*0.019*	8.88	0.26–306.34	0.227	6.95	1.54–1.30	*0.012*
MTLA rating	2.02	1.05–3.87	*0.035*				2.25	1.16–4.35	*0.016*
Log_TBV							0.01	0–1996.50	0.260

## Discussion

The principal finding was the independent association of MTA with early post-stroke cognitive dysfunction in a sample of Nigerian African stroke survivors, apart from the demographic variable of lower educational attainment. In addition, MTA showed significant correlation with WMH, general cognitive performance, executive function, and memory score. Despite a modest sample size, this study is unique in being the first in sub-Saharan Africa to examine neuroimaging correlates of cognitive impairment. Our findings provide robust evidence in support of other previous studies showing the predictive role of MTA in vascular cognitive impairment [7, 8, 30]. Although MTA has often been interpreted as a signature of Alzheimer pathology [8], some recent studies suggest it may also have a vascular basis resulting from cerebral hypoperfusion [31].

Qui et al. [32] recently reported a significant association between aggregated vascular risk factors and reduced hippocampal volume in a cohort of men, while hippocampal neuronal atrophy was found to correlate with PSD in another cohort with insignificant degenerative pathology [33, 34]. There was a trend towards significance in the relationship between aggregated vascular risk factors and MTA as well as in the progression of WMH measures across the cognitive groups. Thus, the relationship between MTA and cognitive impairment in our cohort may suggest a bi-directional causality mediated by cerebral vascular disease. However, the strength of this interpretation is limited by the moderate power (1 − β) = 0.76 of our pilot sample owing to limited availability and high cost of MRI in our study population. Further validation in a bigger cohort studied over time is necessary.

The finding of a significant correlation between MTA and WMH agrees with others [35] and further strengthens the case for a vascular basis in the pathomechanism of MTA, WMH being a surrogate of small vessel disease [12]. We also found a significant association between MTA and WMH, executive function, processing speed and memory in line with previous studies [36, 37]. Similarly, Jokinen et al. found synergistic interactions of MTA, white matter lesions, regional and cortical atrophy on cognitive performance in subjects with small vessel disease in the LADIS study [11]. Our findings, therefore, provide further evidence that global and regional cerebral atrophy, cortico-cortical and cortico-subcortical disconnections and slowing of neural impulse transmission consequent to white matter damage from microvascular pathologies do have robust impact on cognitive processes [4, 12, 13].

Executive dysfunction is an early and prominent feature of vascular cognitive impairment of varying aetiologies and natural history [4, 5]. In previous studies, executive dysfunction had been found to correlate with both WMH [36] and MTA [35, 37] and is thought to further mediate their relationship with memory and visuospatial dysfunction in the context of cerebral vascular disorders.

Surrogates of cognitive reserve include number of years of educational attainment [38] and total brain volume [39]. Our finding of older age and low educational attainment as significant predictors of post-stroke cognitive dysfunction are consistent with previous studies [13, 40–44]. Age is the strongest risk factor for age-associated cerebrovascular and neurodegenerative disorders implicating a likely role for age-related neurodegeneration, synergizing with stroke to cause cognitive impairment and dementia in this cohort [13]. Lower educational attainment is associated with lower cognitive reserve and reduced resilience to dementing brain pathologies [45], especially in the presence of an accompanying reduction in total, cortical or regional brain volume [46]. This pilot study, nonetheless, has several limitations. Though sample size was modest, the significant findings, the first of its kind in sub-Saharan Africa, are worthy of note. The CogFAST—Nigeria project is still in progress in a longitudinal cohort approach with a view to confirming the current findings and unraveling new associations [7]. We assessed white matter changes with the Scheltens’ scale [27]. Generally, visual rating scales are not as sensitive as structural volumetric measures and this may partly explain the lack of statistical significance in the findings of white matter changes in our cohort. Nevertheless, visual rating scales are cost effective, useful in clinical practice and have been proved to attain good reliability and correlation with volumetric measurements [47, 48]. A possibility of selection bias also exists because of our inability to obtain brain MRI for all the available subjects, although we demonstrated that those who had brain imaging did not differ significantly from those who did not have.

## Conclusion

We report an independent association of MTA with early post-stroke cognitive dysfunction in Nigerian African stroke survivors. MTA also showed significant correlation with WMH, general cognitive performance, executive function, and memory score. This study is unique in being the first in sub-Saharan Africa to examine neuroimaging correlates of cognitive impairment and suggests that MTA, which has often been interpreted as a signature of Alzheimer pathology, may have a vascular basis resulting from cerebral hypoperfusion. This study demonstrates feasibility in poor-resourced countries and underscores the importance of early-and long-term sequelae of stroke in survivors [49] that may have implications for the low and middle income countries [50]. Acute and restorative services delivered to stroke survivors will need to be set up in anticipation of a rising number of people with long term motor- and non-motor consequences following stroke, including cognitive impairment. Further studies with larger samples and longitudinal design are needed to unravel more associations.

## Additional files

10.1186/s13104-015-1552-7 Further details on the Cognitive Assessment Battery.
10.1186/s13104-015-1552-7 Tables showing comparisons of subjects with and without Brian MRI and pre-stroke cognitive decline.

## Abbreviations

CRT: choice reaction time
CDR: cognitive drug research
CogFAST: Cognitive Function After STroke
CSID: Community Screening Instrument for Dementia
MRI: magenetic resonance imaging
MTA: medial temporal lobe atrophy
OCSP: Oxford Community Stroke Project Classification
PSD: post-stroke dementia
TBV: total brain volume
VCI: vascular cognitive impairment
vCIND: vascular cognitive impairment no dementia
WMH: white matter hyperintensities
WHO: World Health Organization
