# Supplementary material for: Medial temporal lobe atrophy, white matter hyperintensities and cognitive impairment among Nigerian African stroke survivors
Source: BMC Res Notes. 2015 Oct 30;8:625. doi: 10.1186/s13104-015-1552-7 (PMC4628353; doi:10.1186/s13104-015-1552-7)
Supplement: Supplementary file 1 — 10.1186/s13104-015-1552-7 Further details on the Cognitive Assessment Battery. [file 13104_2015_1552_MOESM1_ESM.docx]

**Supplementary Information Cognitive Assessment Battery**

An ideal neuropsychological battery should be robust, brief, valid, reliable, cost effective, sensitive enough to detect deficits but specific enough to avoid false positives. In addition, it should be easy to administer, be available in multiple forms, have cross cultural capability and have no floor or ceiling effect ([Blake *et al.*, 2002](file:///G:\PhD%20Thesis%20%2030th%20Sept%202013\PhD%20Thesis%20_Revision%20February%202014\Rufus%20AKINYEMI_%20PhD%20Thesis%20_corrected%20version_%20February%202014%20final%2012%2002%202014.docx#_ENREF_53); [Hachinski *et al.*, 2006a](file:///G:\PhD%20Thesis%20%2030th%20Sept%202013\PhD%20Thesis%20_Revision%20February%202014\Rufus%20AKINYEMI_%20PhD%20Thesis%20_corrected%20version_%20February%202014%20final%2012%2002%202014.docx#_ENREF_198)). However, in reality no single tool satisfies all these requirements. New tools being devised, nonetheless, are to aspire to attain these ideal characteristics.

The cognitive assessment tools utilized in this study consisted of the Community Screening Instrument for Dementia (CSID) – cognitive part ([Hall *et al.*, 2000](file:///G:\PhD%20Thesis%20%2030th%20Sept%202013\PhD%20Thesis%20_Revision%20February%202014\Rufus%20AKINYEMI_%20PhD%20Thesis%20_corrected%20version_%20February%202014%20final%2012%2002%202014.docx#_ENREF_205); [Ogunniyi *et al.*, 2000](file:///G:\PhD%20Thesis%20%2030th%20Sept%202013\PhD%20Thesis%20_Revision%20February%202014\Rufus%20AKINYEMI_%20PhD%20Thesis%20_corrected%20version_%20February%202014%20final%2012%2002%202014.docx#_ENREF_409)) the mini-mental state examination (MMSE) ([Folstein *et al.*, 1975](file:///G:\PhD%20Thesis%20%2030th%20Sept%202013\PhD%20Thesis%20_Revision%20February%202014\Rufus%20AKINYEMI_%20PhD%20Thesis%20_corrected%20version_%20February%202014%20final%2012%2002%202014.docx#_ENREF_168)) and the Vascular Neuropsychological Battery (V – NB) ([Hachinski *et al.*, 2006b](file:///G:\PhD%20Thesis%20%2030th%20Sept%202013\PhD%20Thesis%20_Revision%20February%202014\Rufus%20AKINYEMI_%20PhD%20Thesis%20_corrected%20version_%20February%202014%20final%2012%2002%202014.docx#_ENREF_199)). The CSID and the MMSE are tests of general cognitive functioning while the V –NB consists of battery of tests assessing functioning in specific domains of cognition including executive function, language, memory and visuospatial/visuoconstructive domains.

**Community Screening Instrument for Dementia (CSID)**

The CSID is a paper and pencil test of global cognitive performance which adaptability, validity and utility in populations from different cultural, educational and socio-economic backgrounds have been established. From previous validation studies on the CSID, the maximum total cognitive score was 33 while a cut off score of 28.5 was used to define cognitive impairment ([Hall *et al.*, 2000](file:///G:\PhD%20Thesis%20%2030th%20Sept%202013\PhD%20Thesis%20_Revision%20February%202014\Rufus%20AKINYEMI_%20PhD%20Thesis%20_corrected%20version_%20February%202014%20final%2012%2002%202014.docx#_ENREF_205); [Ogunniyi *et al.*, 2000](file:///G:\PhD%20Thesis%20%2030th%20Sept%202013\PhD%20Thesis%20_Revision%20February%202014\Rufus%20AKINYEMI_%20PhD%20Thesis%20_corrected%20version_%20February%202014%20final%2012%2002%202014.docx#_ENREF_409)). It has a sensitivity of 87% and specificity of 83% for the clinical diagnosis of dementia and has been used reliably and widely to assess cognition in the Yoruba speaking population of southwestern Nigeria wherein the present study was conducted. The CSID includes sub-scores for attention, orientation, calculation, short and long term memory, language comprehension and expression, praxis and abstract thinking. A raw score method was used for scoring resulting in score range of 0 – 30 with higher scores indicating better cognitive function.

In the current study, three items were excluded from the CSID. These were two items assessing constructional ability (overlapping circles and interlocking pentagons). They were deleted because the loss of motor function and dexterity in the dominant hand occasioned by stroke could impair performance on these tasks. Moreover, these two items were strongly influenced by education. Also the item assessing the ‘name of local mayor’ was dropped because of confusion between the real names and title names of local mayors in the African setting. Experience had shown that some respondents to this item mentioned the ‘real name’ of the mayor while others mentioned the ‘designated title’ and these were not exactly the same. With this minor revision of the CSID scores, the highest possible score came down to 30 while the cut off for cognitive impairment became 25.5.

**Mini - Mental State Examination (MMSE)**

The Mini - Mental State Examination (MMSE) is a 30 – item test of general cognitive functioning that includes items for assessment of orientation, memory, attention, language and constructional abilities ([Folstein *et al.*, 1975](file:///G:\PhD%20Thesis%20%2030th%20Sept%202013\PhD%20Thesis%20_Revision%20February%202014\Rufus%20AKINYEMI_%20PhD%20Thesis%20_corrected%20version_%20February%202014%20final%2012%2002%202014.docx#_ENREF_168)). In the validated Yoruba version of MMSE, the attention item required the subject to state the days of the week backwards (from Friday to Monday) ([Gureje *et al.*, 1995](file:///G:\PhD%20Thesis%20%2030th%20Sept%202013\PhD%20Thesis%20_Revision%20February%202014\Rufus%20AKINYEMI_%20PhD%20Thesis%20_corrected%20version_%20February%202014%20final%2012%2002%202014.docx#_ENREF_192)). This was a substitute for spelling WORLD backwards in the English version. While a cut -off score of 24 or less is most frequently used to define the presence of dementia in the English version, this was found inappropriate in sample with limited education. A cut-off score of 13 or less for Yoruba-speaking Nigerians with no education and 16 or less for subjects with one or more years of formal education were used in defining possible dementia ([Gureje *et al.*, 1995](file:///G:\PhD%20Thesis%20%2030th%20Sept%202013\PhD%20Thesis%20_Revision%20February%202014\Rufus%20AKINYEMI_%20PhD%20Thesis%20_corrected%20version_%20February%202014%20final%2012%2002%202014.docx#_ENREF_192)).

**Vascular Neuropsychological Battery (V-NB)**

In 2006, the National Institute of Neurological Disorders and Stroke (NINDS) in collaboration with the Canadian Stroke Network (CSN) published the Harmonization Standards for a comprehensive characterization of vascular cognitive impairment ([Hachinski *et al.*, 2006a](file:///G:\PhD%20Thesis%20%2030th%20Sept%202013\PhD%20Thesis%20_Revision%20February%202014\Rufus%20AKINYEMI_%20PhD%20Thesis%20_corrected%20version_%20February%202014%20final%2012%2002%202014.docx#_ENREF_198)). Specific recommendations were made for clinical, neuropsychological, neuroimaging, neuropathological and animal models in the characterization and development of criteria for the definition of vascular cognitive impairment. The neuropsychological sub-section contained recommendations of cognitive tests for 60 –min, 30 – min and 5 - min assessment of the core domains of executive function, language and visuospatial functioning. We, therefore, devised the Vascular Neuropsychological Battery (V –NB) after the NINDS –CSN Harmonization Standards 60 –minute neuropsychological protocol with relevant modifications to ensure adaptability to the language and culture of our predominant Yoruba study population.

The V- NB consists of multiple test items examining specific cognitive domains (executive function, memory/learning, language, visuospatial function/visuoconstructive skills) (Table 2. 1).

Executive function/activation and mental speed were assessed using the category (animal) fluency test, phonemic (letter) fluency test, verbal reasoning and visual reasoning tests. The number of animals listed in the first 15 sec of the animal fluency test provided an assessment of mental speed while all the tests differently assessed mental flexibility and divergent thinking.

The verbal reasoning and visual reasoning tests were adapted from the Cambridge Cognitive Examination (CAMCOG), a cognitive test section of the CAMDEX assessment (Cambridge Mental Disorders of the Elderly Examination) designed to assess a broad range of cognitive functions([Blessed *et al.*, 1991](file:///G:\PhD%20Thesis%20%2030th%20Sept%202013\PhD%20Thesis%20_Revision%20February%202014\Rufus%20AKINYEMI_%20PhD%20Thesis%20_corrected%20version_%20February%202014%20final%2012%2002%202014.docx#_ENREF_55)). It demonstrates excellent psychometric properties and has been used widely in assessing vascular cognitive impairment ([de Koning *et al.*, 2000](file:///G:\PhD%20Thesis%20%2030th%20Sept%202013\PhD%20Thesis%20_Revision%20February%202014\Rufus%20AKINYEMI_%20PhD%20Thesis%20_corrected%20version_%20February%202014%20final%2012%2002%202014.docx#_ENREF_110); [Ballard *et al.*, 2002](file:///G:\PhD%20Thesis%20%2030th%20Sept%202013\PhD%20Thesis%20_Revision%20February%202014\Rufus%20AKINYEMI_%20PhD%20Thesis%20_corrected%20version_%20February%202014%20final%2012%2002%202014.docx#_ENREF_34); [de Koning *et al.*, 2005](file:///G:\PhD%20Thesis%20%2030th%20Sept%202013\PhD%20Thesis%20_Revision%20February%202014\Rufus%20AKINYEMI_%20PhD%20Thesis%20_corrected%20version_%20February%202014%20final%2012%2002%202014.docx#_ENREF_111))

Memory/learning was assessed with the 10- item word list learning test and delayed recall of stick design,([Gureje *et al.*, 1995](file:///G:\PhD%20Thesis%20%2030th%20Sept%202013\PhD%20Thesis%20_Revision%20February%202014\Rufus%20AKINYEMI_%20PhD%20Thesis%20_corrected%20version_%20February%202014%20final%2012%2002%202014.docx#_ENREF_192); [Baiyewu *et al.*, 2005](file:///G:\PhD%20Thesis%20%2030th%20Sept%202013\PhD%20Thesis%20_Revision%20February%202014\Rufus%20AKINYEMI_%20PhD%20Thesis%20_corrected%20version_%20February%202014%20final%2012%2002%202014.docx#_ENREF_29)). The word list learning is a 3 - trial`, 10 – item test with free recall taken after each learning trial and after a brief delay. The total number of words recalled across the three trials make up the total score (range: 0 – 30) while the delayed recall is scored ( 0 -10)`, higher scores indicating better performance. Language was assessed through the 15 – item Boston Naming Test ([Hachinski *et al.*, 2006a](file:///G:\PhD%20Thesis%20%2030th%20Sept%202013\PhD%20Thesis%20_Revision%20February%202014\Rufus%20AKINYEMI_%20PhD%20Thesis%20_corrected%20version_%20February%202014%20final%2012%2002%202014.docx#_ENREF_198)). In a prior validation study of the CERAD battery among Yoruba Nigerians, subjects were requested to name line drawings of common and uncommon objects. Four of the low frequency items from the standard CERAD-NB were replaced with items felt to be more culturally appropriate (i.e. guitar for harmonica, blacksmith tongs for ice cube tongs, mosquito netting for hammock, and 'ayo' (Nigerian board game) for dominoes([Gureje *et al.*, 1995](file:///G:\PhD%20Thesis%20%2030th%20Sept%202013\PhD%20Thesis%20_Revision%20February%202014\Rufus%20AKINYEMI_%20PhD%20Thesis%20_corrected%20version_%20February%202014%20final%2012%2002%202014.docx#_ENREF_192)).

Visuospatial/visuoconstructive functioning was assessed through the Stick Design Test ([Baiyewu *et al.*, 2005](file:///G:\PhD%20Thesis%20%2030th%20Sept%202013\PhD%20Thesis%20_Revision%20February%202014\Rufus%20AKINYEMI_%20PhD%20Thesis%20_corrected%20version_%20February%202014%20final%2012%2002%202014.docx#_ENREF_29)) and the Modified Token Test (IU Token Test) ([Unverzagt *et al.*, 1999](file:///G:\PhD%20Thesis%20%2030th%20Sept%202013\PhD%20Thesis%20_Revision%20February%202014\Rufus%20AKINYEMI_%20PhD%20Thesis%20_corrected%20version_%20February%202014%20final%2012%2002%202014.docx#_ENREF_574); [Akinyemi *et al.*, 2008](file:///G:\PhD%20Thesis%20%2030th%20Sept%202013\PhD%20Thesis%20_Revision%20February%202014\Rufus%20AKINYEMI_%20PhD%20Thesis%20_corrected%20version_%20February%202014%20final%2012%2002%202014.docx#_ENREF_9)) . The Stick Design Test is a non- graphomotor test of visuospatial /visuoconstructive ability. The respondent is requested to use match sticks to reproduce four different graphical shapes with particular attention to the correctness of relative orientation of the match heads. Thereafter the respondent reproduces the four shapes without any cues to assist. The test is particularly useful in older adults with limited formal education ([Baiyewu *et al.*, 2005](file:///G:\PhD%20Thesis%20%2030th%20Sept%202013\PhD%20Thesis%20_Revision%20February%202014\Rufus%20AKINYEMI_%20PhD%20Thesis%20_corrected%20version_%20February%202014%20final%2012%2002%202014.docx#_ENREF_29)). The Modified Token test consists of a piece of laminated paper with a spectrum of squares and circles of varying sizes (small and large) and colours (black, yellow, green and red). The interviewer reads aloud a series of commands that request the subject to point to the figures in different combinations. The test is scored based on correct identification of shapes in the required sequence (range 0 – 24) with higher scores indicating better performance ([Unverzagt *et al.*, 1999](file:///G:\PhD%20Thesis%20%2030th%20Sept%202013\PhD%20Thesis%20_Revision%20February%202014\Rufus%20AKINYEMI_%20PhD%20Thesis%20_corrected%20version_%20February%202014%20final%2012%2002%202014.docx#_ENREF_574)).

Test items from the Cognitive Drug Research (CDR) computerized assessment battery were also included in the V-NB for the evaluation of attention, processing speed and executive function. [The constituent tests included Simple Reaction Time (SRT), Choice Reaction Time (CRT), Digit Vigilance (DV) and Spatial Working Memory (SWM) ([Ballard *et al.*, 2002](file:///G:\PhD%20Thesis%20%2030th%20Sept%202013\PhD%20Thesis%20_Revision%20February%202014\Rufus%20AKINYEMI_%20PhD%20Thesis%20_corrected%20version_%20February%202014%20final%2012%2002%202014.docx#_ENREF_34); [Wesnes, 2002](file:///G:\PhD%20Thesis%20%2030th%20Sept%202013\PhD%20Thesis%20_Revision%20February%202014\Rufus%20AKINYEMI_%20PhD%20Thesis%20_corrected%20version_%20February%202014%20final%2012%2002%202014.docx#_ENREF_597)). The instructions were translated and back –translated from English into Yoruba Language by experienced linguists. Several of these tests were also previously validated and successfully utilized to evaluate cognitive functions in a cohort of Nigerian subjects with Parkinson’s disease ([Akinyemi *et al.*, 2008](file:///G:\PhD%20Thesis%20%2030th%20Sept%202013\PhD%20Thesis%20_Revision%20February%202014\Rufus%20AKINYEMI_%20PhD%20Thesis%20_corrected%20version_%20February%202014%20final%2012%2002%202014.docx#_ENREF_9)). The cognitive assessment battery was pilot - tested on 42 stroke survivors between January and June 2010 in order to further evaluate the feasibility of the methods, acceptance and adaptability in persons living with stroke.

| **Cognitive**  **Domain** | **Test** | **Reference** |
| --- | --- | --- |
| Executive Function | Category (Animal) Fluency Test | *Gureje et al., 1995;* |
| /Activation |  | *Blessed et al, 1991* |
|  |  | *Ballard et al, 2002* |
|  | Phonemic (Letter) Fluency Test | *Blessed et al, 1991* |
|  |  | *Ballard et al, 2002* |
|  | Verbal Reasoning (Similarities Test) | *Blessed et al, 1991* |
|  |  | *Ballard et al, 2002* |
|  | Ideational Fluency Test | *Blessed et al, 1991* |
|  |  | *Ballard et al, 2002* |
| Language/ | Boston Naming Test (2nd version) | *Gureje et al., 1995;* |
| Lexical Retrieval |  | *Akinyemi et al., 2008* |
| Memory/ Learning | Word List Test (Learning, Recall, | *Gureje et al., 1995;* |
|  | Recognition) | *Akinyemi et al., 2008* |
|  | Delayed Recall of Stick Design | *Gureje et al., 1995;* |
|  |  | *Akinyemi et al., 2008* |
| Visuospatial/ | Stick Design Test | *Baiyewu et al., 2005* |
| Visuoconstruction | Modified Tokens Test | *Unverzagt et al., 1999;* |
|  | (IU Token Test) | *Akinyemi et al., 2008* |
| General Cognitive | Community Screening Instrument | *Hall et al, 1993* |
| Functioning | for Dementia (CSID | *Hall et al, 2000* |
|  | Minimental State Examination | *Folstein, 1975* |
|  | (MMSE) | *Gureje et al, 1995* |

**The Vascular Neuropsychological Battery** devised after the 60 –minute Vascular Cognitive Impairment Harmonization Standards – Neuropsychological Protocol proposed by the NINDS – CSN (Hachinski et al, 2006). Multiple test items assessing each cognitive domain were selected in consonance with the recommendations of the Harmonization standards and utility of such tests in previous cognitive evaluations in the environment of the study population.
