# Supplementary material for: Medial temporal lobe atrophy, white matter hyperintensities and cognitive impairment among Nigerian African stroke survivors
Source: BMC Res Notes. 2015 Oct 30;8:625. doi: 10.1186/s13104-015-1552-7 (PMC4628353; doi:10.1186/s13104-015-1552-7)
Supplement: Supplementary file 2 — 10.1186/s13104-015-1552-7 Tables showing comparisons of subjects with and without Brian MRI and pre-stroke cognitive decline. [file 13104_2015_1552_MOESM2_ESM.docx]

**Supplementary Tables 1 and 2:**

**Supplementary Table 1: Comparison of stroke subjects with and without brain MRI**

| Variable | Subjects’ Category | | Test - statistic | p - value |
| --- | --- | --- | --- | --- |
|  | With MRI (n = 58) | Without MRI (n = 85) |  |  |
| Age (mean + SD) | 59.6 (9.6) | 60.8(9.5) | 0.75^b^ | 0.453 |
| Gender [female: n (%)] | 28 (49.1) | 34 (39.5) | 1.28^a^ | 0.302 |
| Years of formal education (mean + SD) | 8.5 (5.7) | 9.9(5.5) | 1.45^b^ | 0.150 |
| Stroke type (CI/ICH/Ind) | 50/8/0 | 64/11/8 | 6.88^a^ | 0.08 |
| Stroke OCSP Classification (LACI/PACI/TACI/POCI) | 23/20/5/2 | 20/33/7/4 | 8.39^a^ | 0.211 |

Abbreviations: CI = Cerebral Infarction; ICH = Intracerebral Haemorrhage; Ind - Indeterminate; LACI = Lacunar Infarct; PACI = Partial Anterior Circulation Infarct; TACI = Total Anterior Circulation Infarct; POCI = Posterior Circulation Infarct. ^a^chi – square test; ^b^students’ t- test

**Supplementary Table 2: Logistic regression model of demographic and imaging determinants of cognitive dysfunction (including and excluding cases with pre –stroke cognitive decline)**

| Variable | Total cohort (N = 58) | | | Total cohort excluding those with pre-stroke cognitive decline (N = 52) | | | |
| --- | --- | --- | --- | --- | --- | --- | --- |
|  | Normal vs (vCIND + PSD) | | | Normal vs (vCIND + PSD) | | | |
| Univariate Analysis | | | | | | | |
|  | OR | 95%CI | p -value | OR | | 95%CI | p - value |
| Age > 60 yrs | **3.97** | **1.30-12.13** | **0.016** | **3.24** | | **1.02 – 10.28** | **0.046** |
| Female gender | 2.34 | 0.81 – 6.74 | 0.116 | 3.091 | | 0.99 – 9.65 | 0.052 |
| Education < 7yrs | **8.52** | **2.58-23.12** | **< 0.001** | **12.50** | | **3.24 -48.26** | **< 0.001** |
| Total WMH score | 1.06 | 1.00-1.13 | 0.073 | 1.05 | | 0.99 – 1.12 | 0.099 |
| Periv WMH score | 1.22 | 0.96-1.56 | 0.102 | 1.14 | | 0.88 – 1.47 | 0.317 |
| Deep WMH score | 1.07 | 0.99-1.15 | 0.094 | 1.06 | | 0.99 – 1.15 | 0.096 |
| Log _ICV | 0.01 | 0.001 – 1144.1 | 0.439 | 0.01 | | 0.00 – 1353.48 | 0.424 |
| Log _TBV | **0.04** | **0.01 – 0.20** | **0.025** | **0.00** | | **0.00 – 0.28** | **0.030** |
| Log _Ven Vol | 18.6 | 0.81 – 429.36 | 0.067 | 12.466 | | 0.52 – 300.87 | 0.120 |
| MTLA Rating | **2.05** | **1.28 – 3.27** | **0.003** | **1.817** | | **1.15 – 2.87** | **0.010** |
| Multivariate Analysis | | | | | | | |
| Nagelkerke R^2^ | R^2^ = 0.490 | | | R^2^ = 0.524 | | | |
| Age > 60 yrs | 0.79 | 0.15 - 4.27 | 0.787 | 0.34 | 0.04 – 2.72 | | 0.311 |
| Female gender | 0.83 | 0.14 - 4.79 | 0.834 | 2.55 | 0.30 – 21.73 | | 0.393 |
| Education < 7yrs | **6.95** | **1.54 – 1.30** | **0.012** | **14.56** | **2.18 – 97.11** | | **0.006** |
| MTLA rating | **2.25** | **1.16 – 4.35** | **0.016** | **2.31** | **1.14 – 4.69** | | **0.021** |
| Log_TBV | 0.01 | 0- 1996.50 | 0.260 | 0.00 | 0.00 – 473003.29 | | 0.522 |
